# Six- and Seven-Membered 1-Oxa-4-Aza-2-Silacyclanes as Possible Correctors of Adaptational Mechanisms

**DOI:** 10.1155/MBD.1998.25

**Published:** 1998

**Authors:** Andrey V. Kurochka, Ol'ga V. Agafonova, Aleksandr S. Losev, Elizaveta A. Mamaeva, Sergey Yu. Bylikin, Vadim V. Negrebetsky, Evgeniya P. Kramarova, Aleksandr G. Shipov, Yuri I. Baukov

**Affiliations:** 1 Russian State Medical University Ostrovityanov St. 1 Moscow 117869 Russia; 2 Research Institute of Pharmacology Russian Academy of Medical Sciences Baltiyskaya St. 8 Moscow 125315 Russia

## Abstract

The biological activity of eight 1-oxa-4-aza-2-silacyclanes with the OSiCH_2_N fragment including 6-membered 2-sila-5-morpholinones (**1–3**) and 4-acyl-2-silamorpholines (**4–6**)and previously unknown 7-membered derivatives of salicylic acid (**7**, **8**) was studied. Compounds **1** and **3–6** show the certain antihypoxic action. Compounds **2** (40 mg/kg), **4** (20 mg/kg), **6** (40 mg/kg), **7** (20 mg/kg) and **8** (40 mg/kg) reduce the physical serviceability of intact animals. Compound **1** (20 mg/kg) influences the physical serviceability in a moderate-positive way on the background of chlorophos-poisoning. Compounds **5–8** displayed protective properties against chlorophos-poisoning at the LD_50_ dose and compounds **2**, **4**, **5**, **7** at the LD_100_ dose. Influence of compounds **1** and **2** on the emotional-research behavior of mice was studied.


### SIX- AND SEVEN-MEMBERED 1-OXA-4-AZA-2-SILACYCLANES AS POSSIBLE CORRECTORS OF ADAPTATIONAL MECHANISMS

Andrey V. Kurochka[1], Ol'ga V. Agafonova[2], Aleksandr S. Losev[2], Elizaveta A. Mamaeva[1], Sergey Yu. Bylikin[1], Vadim V. Negrebetsky[1], Evgeniya P. Kramarova[1], Aleksandr G. Shipov[1] and Yuri I. Baukov*[1]

[1] Russian State Medical University, Ostrovityanov St. 1, Moscow, 117869, Russia
[2] Research Institute of Pharmacology, Russian Academy of Medical Sciences, Baltiyskaya St. 8, Moscow, 125315, Russia

### ABSTRACT

The biological activity of eight 1-oxa-4-aza-2-silacyclanes with the $OSiCH_2N$ fragment including 6-membered 2-sila-5-morpholinones (**1–3**) and 4-acyl-2-silamorpholines (**4–6**) and previously unknown 7-membered derivatives of salicylic acid (**7**,**8**) was studied. Compounds **1** and **3–6** show the certain antihypoxic action. Compounds **2** (40 mg/kg), **4** (20 mg/kg), **6** (40 mg/kg), **7** (20 mg/kg) and **8** (40 mg/kg) reduce the physical serviceability of intact animals. Compound **1** (20 mg/kg) influences the physical serviceability in a moderate-positive way on the background of chlorophos-poisoning. Compounds **5–8** displayed protective properties against chlorophos-poisoning at the $LD_{50}$ dose and compounds **2**, **4**, **5**, **7** at the $LD_{100}$ dose. Influence of compounds **1** and **2** on the emotional-research behavior of mice was studied.

**Keywords**: Organosilicon compounds, 1-oxa-4-aza-2-silacyclanes; Hypoxia-physiopathology; Behavior, animal-drug-effects; Correctors of adaptational mechanisms; Organophosphorous compounds poisoning.

### INTRODUCTION

At present the pentacoordinated organosilicon compounds are the objects of intense investigation because of their unique structures, possible role as the intermediates in $S_N$-Si reactions and biological activity [1–3]. The increased reactivity of such compounds revealed in the course of last years led to investigation of their synthetic applications [2]. In particular, these compounds may be used as precursors for the organoheterosilacyclanes, for example, 1-oxa-4-aza-2-silacyclanes [4, 5].

Taking into account the known physiological significance of silicon and biological activity of organosilicon derivatives (synthesis of glycosaminoglycanes, influence on the calcium, magnesium and aluminium exchange and oxidation metabolism, combination of psychotropic and vazoactive properties and membrane-stabilizing action [3]), it was reasonable to investigate these compounds as the potential correctors of the adaptational mechanisms.

The present article reports the biological activity of eight 1-oxa-4-aza-2-silacyclanes, 6-membered 2-sila-5-morpholinones (**1–3**) and 4-acyl-2-silamorpholines (**4–6**), and previously unknown 7-membered derivatives of salicylic acid (**7**, **8**)** containing the $OSiCH_2N$ fragment. The derivatives of lactic, mandelic, pantoic, γ-hydroxybutyric, isonicotinic, salicylic acids and 2-aminoethanol were chosen due to their known 'pharmacophoric nature'.

### MATERIALS AND METHODS

The IR specra of the compounds were recorded in thin layer and solutions in KBr cells on a Specord IR-75 instrument. The $^1H$ NMR spectra of solutions in $CDCl_3$ were recorded on a Varian XL-400 spectrometer at 400.0 MHz. The chemical shifts were measured using TMS as internal reference.

** For preliminary data on the biological activity of compounds **1** and **2** see [6].

### Synthesis of silacyclanes

The synthesis of 2-sila-5-morpholinones, 2,2,4,6-tetramethyl-2-sila-5-morpholinone (**1**), 2,2,4-trimethyl-6-phenyl-2-sila-5-morpholinone (**2**), 2,2,4-trimethyl-6-(2-trimethylsiloxy-1,1-dimethylethyl)-2-sila-5-morpholinone (**3**) and 4-acyl-2-silamorpholines, 2,2-dimethyl-4-acetyl-2-sila-morpholine (**4**), 2,2-dimethyl-4-isonicotinoil-2-silamorpholine (**5**), 2,2-dimethyl-4-(4-trimethylsiloxybutyril)-2-silamorpholine (**6**) was described previously. The constants of compounds used in biological experiments correspond to the reported values [5].

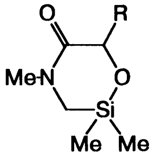

**1–3**

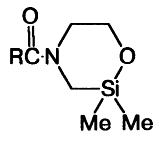

**4–6**

**1**, R = Me; **2**, R = Ph; **3**, R = $CMe_2CH_2OSiMe_3$; **4**, R = Me; **5**, R = $4\text{-}C_5H_5N$; **6**, R = $Me_3SiO(CH_2)_3$

Synthesis of 7-membered silacyclane **7** was carried out in comparison with Scheme 1 using N-methylsalicylamide as initial compound. The amide was transfomed into monosilylated product **9** and then into bissilyl derivative **10** using common routes. Reaction of the latter with chloro(chloromethyl)dimethylsilane (CCMDMS) led to desired silacyclane **7**.

**Scheme 1**

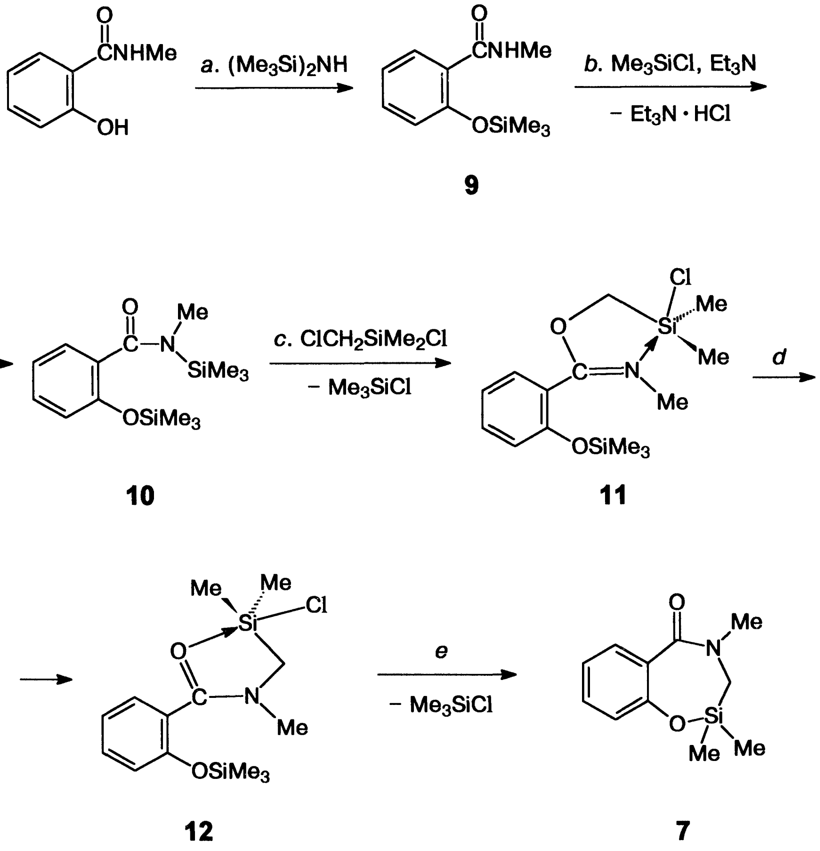

The formation of intermediate O- and N-silylmethylated products **11** and **12** in the course of the reaction was detected by special experiments.

Silacyclane **8** was prepared by the reaction of N-monoethanolsalicylamide with hexamethyldisilazane (HMDS) and subsequent treatment of resulting bissililated product **13** with CCMDMS in the presence of triethylamine (Scheme 2).

**Scheme 2**

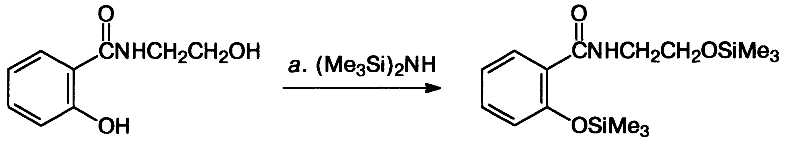

**13**

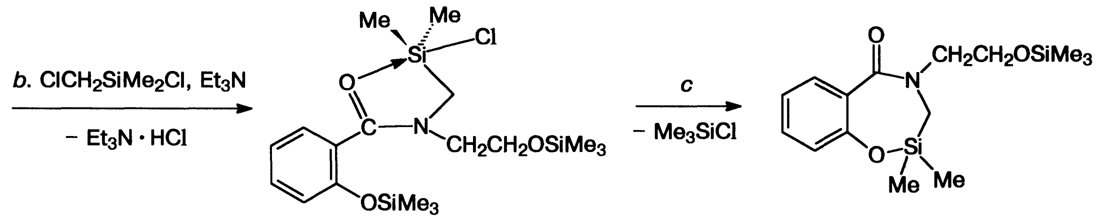

**8**

**2,2,4-Trimethyl-1-oxa-4-aza-2-sila-[6,7]-benzocycloheptan-5-one (7).**

*a. O-Trimethylsilyl-N-methylsalicylamide* (**9**). HMDS (188 ml, 0.9 mol) was added dropwise to 45 g (0.3 mol) of the N-methylamide of salicylic acid. The reaction mixture was refluxed under vigorous stirring for 2 h. After removing the excess of HMDS *in vacuo* the residue was fractionated and 58 g (95%) of the desired product (**9**) were obtained. B. p. 160–161°C (10 mm Hg), $n_D^{20}$ 1.5150. IR spectrum (ν, $cm^{-1}$): 1669 (C=O). NMR $^1H$ spectrum (δ, ppm, $CDCl_3$): 0.33 s (9H, $Me_3Si$), 2.94 d (3H, MeN), 6.2–6.7 m (4H, $C_6H_4$), 8.04 d (1H, NH). Anal. Calcd for $C_{11}H_{17}NO_2Si$, %: C 59.13, H 7.67. Found, %: C 59.31, H 7.56.

*b. O,N-bis(trimethylsilyl)-N-methylsalicylamide* (**10**). Trimethylchlorosilane (TMCS, 36.7 g, 0.34 mol) was added dropwise to a solution of 47 g (0.23 mol) of compound **9** and 52 ml (0.34 mol) of triethylamine in 100 ml of diethyl ether. The reaction mixture was refluxed under vigorous stirring for 3 h. The precipitated salt was filtered out, the solvent was removed *in vacuo*. After the fractionation of the residue 50 g (78%) of compound **10** were obtained. B. p. 143–144°C (10 mm Hg), $n_D^{20}$ 1.5145. IR spectrum (ν, $cm^{-1}$): 1636 (C=O). $^1H$ NMR spectrum (δ, ppm, $CDCl_3$): 0.35 broad (18H, $Me_3SiO$+$Me_3SiN$), 2.78 s (3H, MeN), 6.78–7.48 m (4H, $C_6H_4$). Anal. Calcd for $C_{14}H_{25}NO_2Si_2$, %: C 56.88, H 8.52. Found, %: C 57.21, H 8.18.

*c. One-pot synthesis of silacyclane* (**7**). The mixture of 10 g (36 mmol) of amide **10** and 3 g (36 mmol) of CCMDMS was heated until the complete elimination of TMCS (64–66°C, 9.2 ml, 100%). After the fractionation of the residue 6.9 g (86%) of silacyclane **7** were obtained. B. p. 178–180°C (7 mm Hg). IR spectrum (ν, $cm^{-1}$): 1624 (NCO). $^1H$ NMR spectrum (δ, ppm, $CDCl_3$): 0.38 s (6H, $Me_2Si$), 2.89 s (2H, $CH_2$), 3.21 s (3H, MeN), 6.9–7.8 m (4H, $C_6H_4$). Anal. Calcd for $C_{11}H_{15}NO_2Si$, %: C 59.69, H 6.38, Si 12.69. Found, %: C 59.24, H 6.90, Si 12.05. Mol. weigth (cryoscopy, $C_6H_6$): Calcd 221.38; found 222.

*d. Evidence for the intermediate (N—Si)-chelated O-(dimethylchlorosilylmethy)-N-methyl-salicylamide (**11**) and (O—Si)-chelated O-trimethylsilyl-N-(dimethylchlorosilylmethyl)-N-methyl-salicylamide (**12**).* CCMDMS (1.6 g, 11 mmol) was added dropwise to the solution of 2.25 (10 mmol) of amide **10** in 5 ml of diethyl ether. In 5 min the presence of an intense band of

O-silylmethylated product (1669 $cm^{-1}$) along with the bands at 1636 and 1597 $cm^{-1}$ corresponding to the compound **10** and the N-silylmethylated product **12**, respectively, was detected in the IR spectrum of the reaction mixture. Afterwards the quick decrease of the bands at 1669 and 1639 $cm^{-1}$ along with a moderate increase of the band at 1597 $cm^{-1}$ was observed. In 30 min only the product of N-silylmethylation **12** was detected in the IR spectrum of the mixture. After the removal of TMCS and ether *in vacuo* at room termperature 2.6 g (79%) of the chloride **12** were obtained (yellow oil). IR spectrum (ν, $cm^{-1}$): 1597, 1500 (NCO). $^1$H NMR spectrum (δ, ppm, $CDCl_3$): 0.26 s (6H, $Me_2Si$), 0.36 s (9H, $OSiMe_3$), 2.99 s (3H, MeN), 3.04 s (2H, $CH_2$), 6.8–7.7 m (4H, $C_6H_4$).

**2,2-Dimethyl-4-(2-trimethylsiloxyethyl)-1-oxa-4-aza-2-sila-[6,7]-benzocycloheptan-5-one (8).**

*a. O,O'-Bis(trimethylsilyl)salicylmonoethanolamide* (**13**). A mixture of ethyl salicylate (152 g, 1 mol) and monoethanolamine (61 g, 1 mol) was refluxed with removal of ethanol for 2 h. After recrystallization of the residue from ethyl acetate 107.5 g (65%) of the monoethanolamide of salicylic acid were obtained. IR spectrum (ν, $cm^{-1}$): 1630, 1560 (NHCO). The latter was refluxed with 150 ml of HMDS for 2 h. After the fractionation 58 g (54%) of compound **13** were obtained. B. p. 172–174°C (5 mm Hg), $n_D^{20}$ 1.4395. IR spectrum (ν, $cm^{-1}$): 1645, 1510 (NCO). $^1$H NMR spectrum (δ, ppm, $CDCl_3$): 0.10 s (9H, $Me_3SiO$), 0.35 s (9H, $Me_3SiN$), 3.55 dt (2H, $NCH_2$, $^3J_{HH}$ 7.0 Hz, 7.0 Hz), 3.73 t (2H, $OCH_2$, $^3J_{HH}$ 7.0 Hz), 6.8–8.2 m (4H, $C_6H_4$), 8.15 broad (1H, NH). Anal. Calcd for $C_{15}H_{27}NO_3Si_2$, %: C 55.34, H 8.36. Found, %: C 54.93, H 8.12.

*b. Silacyclane* (**8**). CCMDMS (18 g, 0.13 mol) was added dropwise for 30 min to the solution of 34 g (0.1 mol) of compound **13** and 14.5 g (0.13 mol) of triethanolamine in 150 ml benzene. The mixture was refluxed for 3 h and allowed to stand overnight. The precipitate was filtered out, the solvent was removed *in vacuo*. After the fractionation of the residue 23 g (72%) of silacyclane **8** were obtained. B. p. 166–167°C (1 mm Hg), $n_D^{20}$ 1.5110. IR spectrum (ν, $cm^{-1}$): 1616 (C=O). $^1$H NMR spectrum (δ, ppm, $CDCl_3$): 0.09 s (9H, $Me_3Si$), 0.31 s (6H, $Me_2Si$), 2.92 s (2H, $NCH_2Si$), 3.68 t (2H, $NCH_2$, $^3J_{HH}$ 7.0 Hz), 3.84 t (2H, $OCH_2$, $^3J_{HH}$ 7.0 Hz), 6.8–7.6 m (4H, $C_6H_4$). Anal. Calcd for $C_{15}H_{25}NO_3Si$, %: C 55.68, H 7.79, Si 17.36. Found, %: C 55.77, H 7.69, Si 17.38.

### Biological study

The experiments were carried out on white alley mice, hares, weight 16–24 g. All compounds were injected as water emulsion in 'twin-80' intraperitoneal in the given range of doses before an hour to the beginning of the registration of parameters. The animals of control groups were injected with adequate volumes of salt infusion. The quantity of animals in each group varied from 6 to 15.

For data processing the basic methods of parametrical and unparametrical statistics (*t*-criterion Student, discriminant function analysis, cluster analysis, correlation analyses) were used.

**Acute toxicity** of compounds was defined by the express train-method of V. Prozorovsky [7].

**Acute hypobaric hypoxia** (AHBH) was simulated in the flowing hypobaric chamber (absorber of carbon dioxide, the 30% solution of KOH at 18–22°C) by 'raising' of the animals on the height of 11000 m with the average speed of 50 m/sec. The survival rate of animals and the time of their death (reserve time) were estimated [8].

**Acute immersion cooling** (AIC) was simulated by compulsory navigation without loading in ice water (3.5–4.0 °C). The duration of life of the animals had been estimated until the clonic spasms and characteristic 'pose of capuchin' appeared.

**Physical serviceability** (PS) was estimated on duration of floating in water at 28–29 °C with a cargo. It was attached to the basis of a tail, equal to 5% from weight of a body [9].

**The preventive maintenance of acute organophosphorus compounds poisoning** (OPhC). The compounds were injected an hour before the chlorophos poisoning (1% solution, subcutaneosly in doses $LD_{50}$ and $LD_{100}$). The survival rate had being analyzed within a day.

**Therapeutic effect of the compounds at an acute organophosphorus compounds poisoning** (OPhC). The compounds were injected an hour before the chlorophos poisoning (1% solution; subcutaneosly in doses $LD_{50}$ and $LD_{100}$). The survival rate had being analyzed within a day.

**The influence of compounds on individual behavior**. The psychotropic effects of the compounds on individual rough-research behavior were studied on the model called 'an open field'. The 'open field' for mice was composed of a chamber by the size 40x40x40 sm, painted in white colour and closed by a transparent cover. The floor of the chamber was lined on squares 10x10 sm with a round aperture ('mousehole') with a diameter of 3 sm at the centre of each square. The research was carried out at electrical illumination by a glow-lamp with capacity 100 W, located on distance 1.5 m from the center of the field.

**Behavior of each animal** was estimated individually. A mouse was placed in the left corner of the chamber. The complete structure of behavior had being registered for 4 minutes. It included the following elements: smelling, moving, movement on a place, condition in a rule sitting, looking in 'mousehole', vertical rule (stance with an emphasis, vertical stance), grooming, freezing, and also the number of defecations and micturitions. It is necessary to add that not only the expression of elements of behavior was estimated but also the probabilities of transitions from one condition into the other were estimated [13].

The influence of compounds on metabolism was described by us previously [6].

### RESULTS AND DISCUSSION

### *Chemical Aspects*

The general synthetic approach to 1-oxa-4-aza-2-silacyclanes consists of the next steps. The initial compounds are the monosubstituted amides of carboxylic acids containing the additional hydroxylic group at the acid radical or/and at the substituent at the N atom. On the first stage (Schemes 1,2, reactions *a*) this group should be protected by the transformation into the corresponding O-trimethylsilyl derivative. One of possible ways for the next stage is the reaction of the latter with CCMDMS in the presence of a base (*amination method*) leading to the unstable (O—Si)-chelated products of N-chlorodimethylsilylmethylation which contain the pentacoordinated silicon atom. The thermal decomposition of these compounds with the elimination of $Me_3SiCl$ in the course of fractionation gives the desired silacyclanes. By this method, the known 2-sila-5-morpholinones **1–3**, 4-acyl-2-silamorpholines **4–6** and the derivative of salicylic acid **8** (Scheme 2) were prepared.

The higher yields of desired silacyclanes **1–6** and **8** may be obtained without the isolation of unstable pentacoordinated organosilicon chlorides. The existence of such intermediate compounds was detected by the IR and NMR monitoring [5]. IR specra of impure chlorides show two bands at 1600–1605 and 1515–1520 $cm^{-1}$ of chelate $C(O)NCH_2Si$ fragment [10]. Intramolecular interaction O→Si in these compounds was also confirmed by the upfield shift of signals in the $^{29}Si$ NMR spectra (up to –30 ÷ –40 ppm), which testifies to the presence of pentacoordinated silicon atom [1, 11].

Another way includes the transformation of the O-silylated product **9** under rather drastic conditions into the corresponding bis-O,N-trimethylsilylmethylated product **10** (Scheme 1, reaction *b*) following by the reaction with CCMDMS (reaction *c*, *transsilylation method*).

The formation of intermediate O-silylmethylated product **11** and its subsequent isomerization into the N-silylmethylated product **12** (reactions *c*, *d*) were confirmed by the IR specroscopy.

The general scheme of the reactions of trimethylsilyl derivatives of amides and lactams with CCMDMS includes the intermediate product of transsilylation [12]. However, in the case of bis-O,N-trimethylsilyl derivative **10** the corresponding product of could not be detected by the IR spectroscopy and so it was excluded from scheme 1.

### *Pharmacology*

The definition of acute toxicity of silacyclanes **1–8** on the first investigation phase revealed their low toxicity ($LD_{50}$ 400–500 mg/kg) [14] and allowed to choose the acceptable doses for the following experiments. Futher the doses equal 1/40–1/10 $LD_{50}$ (10, 20, 40 mg/kg) were used.

The study of antihypoxic activity of silacyclanes **1–8** (Table I) showed that compounds **1** (20 mg/kg), **3** (40 mg/kg), **4** (20 mg/kg), **5** (20 mg/kg) and **6** (20 mg/kg) increase the time of life of mice in AHBH condition with different efficiency. Compounds in doses of 10 mg/kg did not affect the time

**Table I.** Biological activity of silacyclanes **1—8**

| Compound | Dose (mg/kg) | Models: AHBH[a], % to control group (P) | PS[b], % to control group (P) | AIC[c], % to control group (P) | PS/OPhC[d], % to control group (P) | % to control group (P) |
|---|---|---|---|---|---|---|
| **1** | 0 | 100±24 | 100±17 | 100±9 | 25±5 (<0.001) | 100±17 |
| | 20 | 185±34 (<0.05) | 123±28 (>0.5) | 88±8 (<0.3) | 34±4 (<0.01) | 135±16 (<0,1 ) |
| | 40 | 160±42 (<0.3) | | 95±13 (<0.5) | | |
| **2** | 0 | 100±24 | 100±17 | 100±7 | 25±5 (<0.001) | 100±17 |
| | 20 | 69±38 (<0.5) | | 116±11 (<03) | | |
| | 40 | 138±5 (<0.5) | 54±8 (<0.05) | 109±9 (<0.5) | 31±8 (<0.001) | 123±29 (>0.5) |
| **3** | 0 | 100±11 | 100±17 | | 25±5 (<0.001) | 100±17 |
| | 20 | 139±20 (<0.2) | 100±21 (>0.5) | | | |
| | 40 | 140±18 (<0.2) | | | 24±6 (<0.001) | 94±25 (>0.5) |
| **4** | 0 | 100±11 | 100±17 | | 25±5 (<0.001) | 100±17 |
| | 20 | 158±11 (<0.01) | 47±12 (<0.05) | | 23±7 (<0.02) | 109±34 (>0.5) |
| | 40 | 63±10 (<0.1) | | | | |
| **5** | 0 | 100±44 | 100±17 | | 25±5 (<0.001) | 100±17 |
| | 20 | 331±163 (0.1<P<0.25) | 84±19 (>0.1) | | 21±4 (<0.01) | 101±20 (>0.5) |
| | 40 | 58±9 (<0.5) | | | | |
| **6** | 0 | 100±11 | 100±17 | | 25±5 (<0.001) | 100±17 |
| | 20 | 160±9 (<0.01) | | | 19±4 (<0.001) | 75±15 (<0.5) |
| | 40 | 847±421 (0.05<P<0.1) | 62±8 (0.05<P<0.1) | | | |
| **7** | 0 | 100±44 | 100±17 | | 25±5 | 100±17 |
| | 20 | 369±29 (<0.5) | 42±8 (<0.02) | | 15±2 (<0.001) | 74±7 (>0,1) |
| | 40 | 197±131 (<0.5) | | | | |
| **8** | 0 | 100±44 | 100±17 | | 25±5 | 100±17 |
| | 20 | 64±9 (<0.5) | | | | |
| | 40 | 203±130 (<0.5) | 63±4 (<0.05) | | 18±2 (<0.001) | 84±10 (>0,1) |

The note to Table. I

[a] Stability to acute hypobaric hypoxia, [b] Physical serviceability of mice in natatory test, [c] Stability to acute immersion cooling, [d] Physical serviceability of mice in natatory test on the background of chlorophos poisoning ($0.5LD_{50}$).

of life. The analysis of experimental results allows to assume the presence of certain antihypoxic activity of compounds **1**, **3–6**. The observed antihypoxic effects are comparable with the effects of (2-oxo-1-pyrrolidinyl)acetamide (nootropil) or sodium $\gamma$-hydroxybutanoate in effective doses [8]. Compounds **7** and **8** showed no antihypoxic activity.

Silacyclanes **2** (40 mg/kg), **4** (20 mg/kg), **6** (40 mg/kg), **7** (20 mg/kg) and **8** (40 mg/kg) reduce the PS while compounds **1** (20 mg/kg), **3** (40 mg/kg) and **5** (20 mg/kg) do not affect it.

The effects of compounds **1** and **2** were investigated using the model of acute immersion cooling. They showed no influence on resistance of mice to AIC (see Table I).

The influence of silacyclanes **1–8** on PS of mice in natatory test with a cargo was investigated on the background of chlorophos poisoning (0.5 $LD_{50}$). The duration of natatory was increased after injection of compound **1** (20 mg/kg). Compounds **2** (40 mg/kg), **3** (40 mg/kg), **4** (20 mg/kg) and **5** (20 mg/kg) did not influence on it while the compounds **6** (20 mg/kg), **7** (20 mg/kg) and **8** (40 mg/kg) reduced this parameter (Table I).

It is known that the adaptation of organism to AHBH and OPhC compound poisoning has common mechanism [15]. Therefore, the combination in system screening of models AIC and OPhC poisoning was of interest as a confirmation of direct correlation between frigoprotective properties of compounds and their influence on PS. Besides, it was necessary to expect the return dependence between antihypoxic activity and stimulating action (on the AHBH and OPhC model), and also the OPhC-protective action and stimulating action.

The results of study OPhC-protective properties of silacyclanes **1–8** are summarized in Table II. At chlorophos-poisoning in a dose $LD_{50}$ protective properties were displayed by compounds **5–8**; at a dose $LD_{100}$ by compounds **2**, **4**, **5**, **7**.

**Table II**. A preventive effect of the compounds on dynamics survival rate of animals within a day after the injection of chlorophos ($LD_{50}$ or $LD_{100}$).

| Compound | Dose mg/kg | Survival within a day (min) 10 | 20 | 30 | 40 | 50 | 60 | 24 hours |
|---|---|---|---|---|---|---|---|---|
| chlorophos | $LD_{50}$ | 8/8 | 8/8 | 5/8 | 2/8 | 2/8 | 2/8 | 2/8 |
| **1** | 20 | 6/6 | 6/6 | 4/6 | 2/6 | 2/6 | 2/6 | 2/6 |
| **2** | 40 | 6/6 | 5/6 | 2/6 | 2/6 | 2/6 | 2/6 | 2/6 |
| **3** | 40 | 6/6 | 6/6 | 6/6 | 4/6 | 4/6 | 4/6 | 0/6 |
| **4** | 20 | 6/6 | 6/6 | 5/6 | 2/6 | 2/6 | 2/6 | 2/6 |
| **5** | 20 | 6/6 | 6/6 | 5/6 | 5/6 | 5/6 | 5/6 | 5/6 |
| **6** | 20 | 6/6 | 6/6 | 5/6 | 3/6 | 3/6 | 3/6 | 3/6 |
| **7** | 20 | 6/6 | 6/6 | 4/6 | 4/6 | 4/6 | 4/6 | 4/6 |
| **8** | 40 | 6/6 | 6/6 | 6/6 | 5/6 | 5/6 | 5/6 | 5/6 |
| chlorophos | $LD_{100}$ | 8/8 | 4/8 | 0/8 | 0/8 | 0/8 | 0/8 | 0/8 |
| **1** | 20 | 6/6 | 6/6 | 4/6 | 1/6 | 1/6 | 1/6 | 1/6 |
| **2** | 40 | 6/6 | 5/6 | 3/6 | 2/6 | 2/6 | 2/6 | 2/6 |
| **3** | 20 | 6/6 | 3/6 | 3/6 | 1/6 | 1/6 | 1/6 | 1/6 |
| **4** | 20 | 6/6 | 6/6 | 5/6 | 5/6 | 4/6 | 4/6 | 4/6 |
| **5** | 20 | 6/6 | 6/6 | 5/6 | 2/6 | 2/6 | 2/6 | 2/6 |
| **6** | 40 | 6/6 | 6/6 | 0/6 | 0/6 | 0/6 | 0/6 | 0/6 |
| **7** | 20 | 6/6 | 6/6 | 3/6 | 2/6 | 2/6 | 2/6 | 2/6 |
| **8** | 40 | 6/6 | 6/6 | 4/6 | 1/6 | 1/6 | 1/6 | 1/6 |

In order to reveal an ethological effect of the compounds and to define the most important parameters the profound screening of compounds **1** and **2** was carried out using the 'open field' technique. Behavioral patterns and their communications were registered (Table III, Fig. 1).

It was found that 2-sila-5-morpholinones **1** and **2** do not render the expressed negative action on the spectrum of individual behavior.

**Table III**. The influence of compounds **1** and **2** on elements of behavior in 'open field'.

| Elements of behavior | Expression of elements of behavior, sec | | | | |
|---|---|---|---|---|---|
| | Control, n = 10 | 1, n = 10 | * | 2, n = 10 | * |
| Smelling | 97.6±4.2 | 103.7±2.9 | Δ | 98.1±7.2 | |
| Moving | 35.3±7.4 | 34.3±6 | | 34.6±11.2 | |
| Sitting | 27.4±9 | 27.5±8.4 | | 26.2±10.6 | |
| Looking in "mousehole" | 51.9±2.2 | 45.4±4.7 | ∇ | 31.0±5.1 | ∇∇ |
| Movement on a place | 16.0±3.3 | 16.2±2.0 | | 20.9±2.6 | Δ |
| Grooming | 8.1±3.6 | 7.4±2.6 | ∇ | 5.5±2.2 | ∇ |
| Stance with an emphasis | 4.3±1.4 | 3.2±1.1 | ∇ | 0.0±0 | ∇∇ |
| Vertical stance | 1.4±0.5 | 0.3±0.2 | ∇∇ | 2.6±1.2 | Δ |
| Freezing | 0.00±0 | 1.9±1.9 | Δ | 27.4±14.5 | Δ |
| Defecations | 0.9±0.2 | 1.1±0.5 | | 0.6±0.3 | |
| Micturitions | 0.00±0 | 0.00±0 | | 0.00±0 | |

* — the parameter is increased; — the parameter decreases; ∇∇ — the parameter decreases authentically

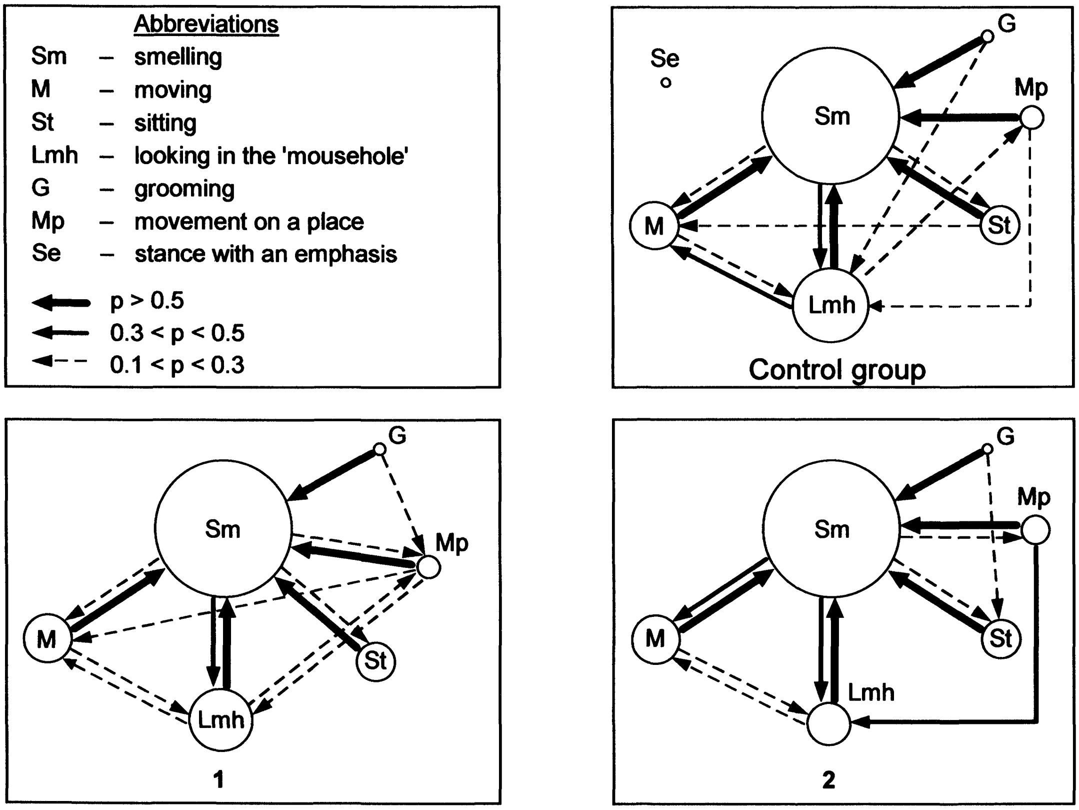

**Fig. 1**. Graph-structure of the mice behavior in “open field” in an hour after the introduction of compounds **1** and **2**. Circles are the behavioral patterns (the diameter corresponds to the degree of expression), arrows show the probability of transitions of behavior elements (the thickness corresponds to statistical meanings of the probability).

At the same time, the discriminant function analysis and the cluster analysis showed that compounds **1** and **2** are different to each other and to the control in influence on the structure of behavior. The parameters of emotional-research behavior of animals (stance with an emphasis, vertical stance, grooming, looking in 'mousehole') are the differentiating attributes (general probability of distinction is 0.8). It is considered as sedative-tranquilizing action which is useful at various kinds of stress and extreme influences.

The study of 1-oxa-4-aza-2-silacyclanes proved them to be perspective objects for the investigation of means with psychotropic activity, directed on preventive maintenance stress prevention, and also with the properties of metabolic proof-readers of hypoxia, i. e. the compounds with actoprotective activity. It was demonstrated on a limited quantity of biological material within the framework of the submitted system screening.

This work was supported by INTAS (Project No. 93-1411) and the Russian Foundation for Basic Research (Project No. 96-03-32718).

**References**

1. S. N. Tandura, M. G. Voronkov, and N. V. Alekseev, *Top. Curr. Chem.* Berlin; etc.: Springer-Verlag, 1986, **131**, 99; M. G. Voronkov, V. A. Pestunovich, and Yu. I. Baukov, *Metalloorg. Khim.* (in Russian), 1991, **4**, 1210 [*Organomet. Chem. USSR*, 1991, **4**, 917 (Engl. Transl.)].
2. C. Chuit, R. J. P. Corriu, C. Reye, and J. C. Young, *Chem. Rev.*, 1993, **93**, 1371.
3. M. G. Voronkov, G. I. Zelchan, E. Lukevitz. Silicium und Leben. Berlin, Akademie—Verlag, 1975, 370 p.; M. G. Voronkov, *Top. Curr. Chem.* Berlin; etc.: Springer-Verlag, 1979, **84**, 77–135.
4. Yu. I. Baukov, A. G. Shipov, E. P. Kramarova, O. B. Artamkina, E. A. Mamaeva, and S. Yu. Bylikin, *Xth International Symposium on Organosilicon Chemistry, August 15–20, 1993,* Poznan, Poland, 1993, 131; A. G. Shipov, E. P. Kramarova, S. Yu. Bylikin, E. A. Mamaeva, G. S. Zaitseva, V. N. Sergeev, and Yu. I. Baukov, *Zh. Obshch. Khim.* (in Russian), 1993, **63**, 1195.
5. Yu. I. Baukov, A. G. Shipov, E. P. Kramarova, E. A. Mamaeva, O. A. Zamyshlyaeva, N. A. Anisimova, and Vad. V. Negrebetsky, *Zh. Organ. Khim.* (in Russian), 1996, **32**, 1259.
6. E. A. Mamaeva, O. V. Agafonova, Vad. V. Negrebetsky, A. G. Shipov, Yu. I. Baukov, and A. S. Losev, *Khim.-pharm. Zh.* (in Russian), 1994, No. 6, 26.
7. V. B. Prozorovsky, M. P. Prozorovskaya, and V. M. Demchenko, *Pharmacology and Toxicology* (in Russian), 1978, 497.
8. Methodical recommendations on experimental investigation of compounds for clinical investigation as antihypoxic remedies., Ed. L. D. Luk'yanova, Moscow, 1990, 87 p. (in Russian).
9. M. L. Rylova, Methods of investigation of chronic action of enviromental agressive factors in experiments , Medicine, Leningrad, 1964, 88 p. (in Russian).
10. R. W. Hillyard, C. M. Ryan, and C. H. Yoder, *J.Organometal. Chem.* 1978, **153**, 369; Yu. I. Baukov, E. P. Kramarova, A. G. Shipov, G. I. Oleneva, O. B. Artamkina, A. I. Albanov, M. G. Voronkov, and V. A. Pestunovich, *Zh. Obshch. Khim.* (in Russian), 1989, **59**, 127.
11. E. A. Williams, *NMR spectroscopy of organosilicon compounds*, In *The chemistry of organic silicon compounds,* Eds. S. Patai, Z. Rappoport, Chichester, Willey, 1989. Ch. 8. 537–540.
12. I. D. Kalikhman, A. I. Albanov, O. B. Bannikova, L. I. Belousova, M. G. Voronkov, V. A. Pestunovich, A. G. Shipov, E. P. Kramarova, and Yu. I. Baukov, *J. Organometal. Chem.* 1989. **361**. 147.
13. A. M. Chirkov, S. K. Chirkova, I. S. Voyt, and A. L. Dinsburg., *Sechenov Physiological Journal*, 1993, **79**, 25 (in Russian).
14. S. D. Zaugol'nikov, M. M. Kochanov, A. O. Loyt, and Stavchansky, Express methods of toxicity determination and safety of chemical compounds, Medicine, Leningrad, 1978, 184 p. (in Russian).
15. A. F. Fesyuk, A. S. Losev, I. S. Morosov, A. I. El'kin, and A. V. Kurochka, *Pharmacological correction of hypoxic conditions.* In *Materials of IIth All-Union Conference*, Grodno, Russua, 1991, Ch. 1, 121 (in Russian).

**Received: July 22, 1997 - Accepted: August 14, 1997 -**
**Received in revised camera-ready format: January 13, 1998**

